# Supplementary material for: Assessment of adult structural plasticity in Drosophila neurons
Source: Open Biol. 2026 Sep 9;16(9):260120. doi: 10.1098/rsob.260120 (PMC13561074; doi:10.1098/rsob.260120)
Supplement: Supplementary file 1 [file rsob.260120.zip › rsob.260120/rsob260120_si_001.pdf]

Assessment of adult structural plasticity in *Drosophila* neurons

Micaela Rodriguez-Caron[1], Francisco J. Tassara[1,2], Juan I. Ispizua[1,3], Christian M. Carpio-Romero[1,2], M. Fernanda Ceriani[#1]

[1] Laboratorio de Genética del Comportamiento. Fundación Instituto Leloir, IIB-BA/CONICET. Buenos Aires, Argentina

[2] Facultad de Ciencias Exactas y Naturales, Universidad de Buenos Aires, Buenos Aires 1428, Argentina.

[3] Current address: Department of Neurobiology and Biophysics, University of Washington, Seattle, WA, USA

[#] Corresponding author

Open Biology, DOI: 10.1098/rsob.260120

Supplementary information

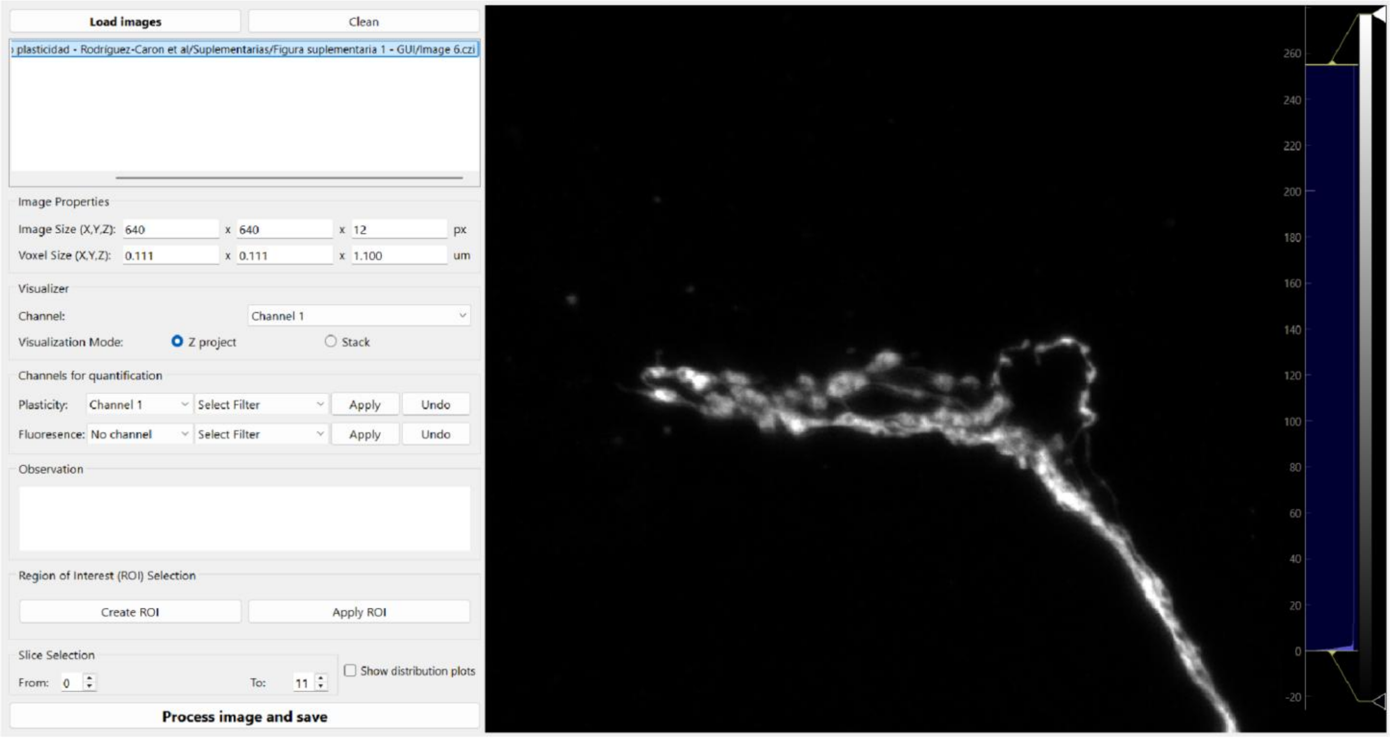

Supplementary Figure 1. *MorphoScope* graphical user interface. The *MorphoScope* interface was developed to facilitate the complexity quantification of multiple terminal images based on the algorithm originally described in Petsakou et al. (1). The interface is divided into functional modules, beginning on the left with “Load images” for selecting input files (.tiff, .czi, or .lsm), followed by “Image properties”, which displays metadata like image and voxel sizes.

The “Visualizer” controls the displayed channel on the right and selects between Z-projection or the full Z-stack visualization, while the “Channels for quantification” module specifies which channel is used for plasticity and which is used for fluorescence quantification, and also allows for filter application. Before processing, the user can define a note in “Observation”. Then, sets the area of interest using “ROI selection” and specifies the range of Z-planes via “Slice selection” to encompass the full terminal. Finally, quantification is executed by clicking “Process image and save” with results automatically added to an Excel file generated upon image loading. For further details, see https://github.com/FranTassara/MorphoScope.

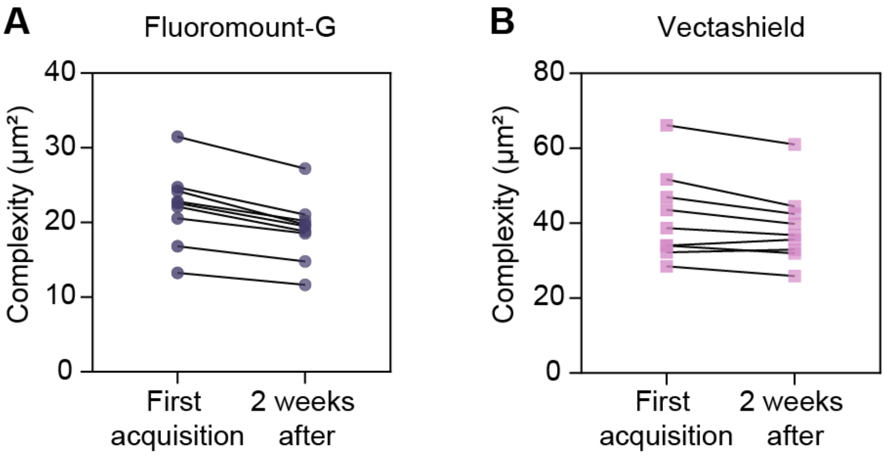

Supplementary Figure 2. Stability of neuronal complexity over time in different mounting media. Comparison of the terminal complexity measured on the day after mounting (first acquisition) and two weeks after for samples mounted in Fluoromount-G **(A)** and Vectashield **(B)**. Individual lines connect the same terminal re-acquired over time.

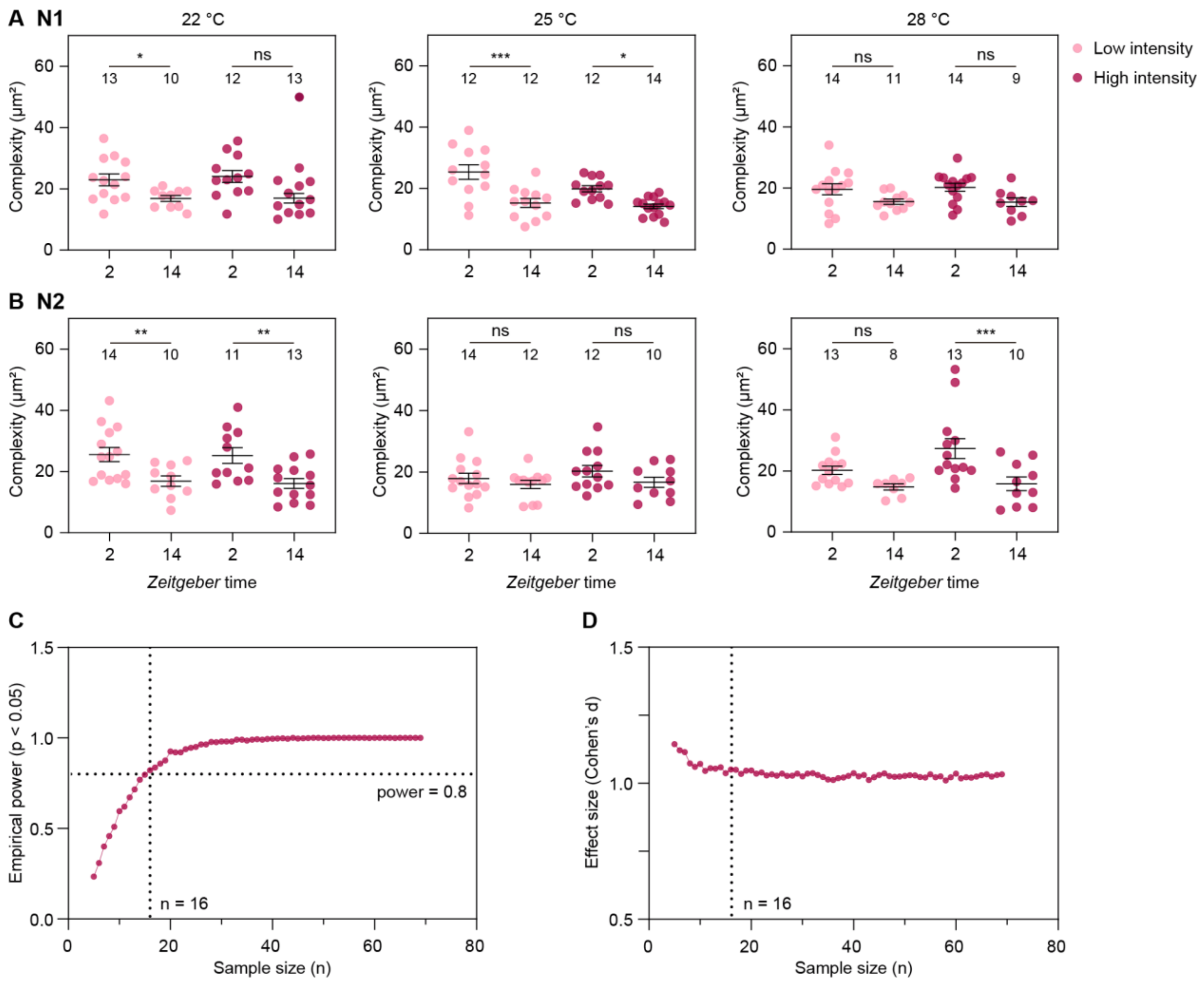

Supplementary Figure 3. Sample size calculation. (A and B) Structural complexity quantification at different temperatures and light intensities. The panels display the two individual experiments that were shown pooled in **Figure 3B**. **(C)** Empirical power as a function of sample size. It shows the proportion of simulations yielding a statistically significant result ($p < 0.05$) across increasing sample sizes. The horizontal dotted line marks 80% power, and the vertical one indicates the sample size at which this threshold is achieved ($n = 16$). **(D)** Estimated effect size (Cohen's d) as a function of sample size. It depicts the mean estimated effect size from repeated simulations across sample sizes. The vertical dotted line again marks $n = 16$ for reference.

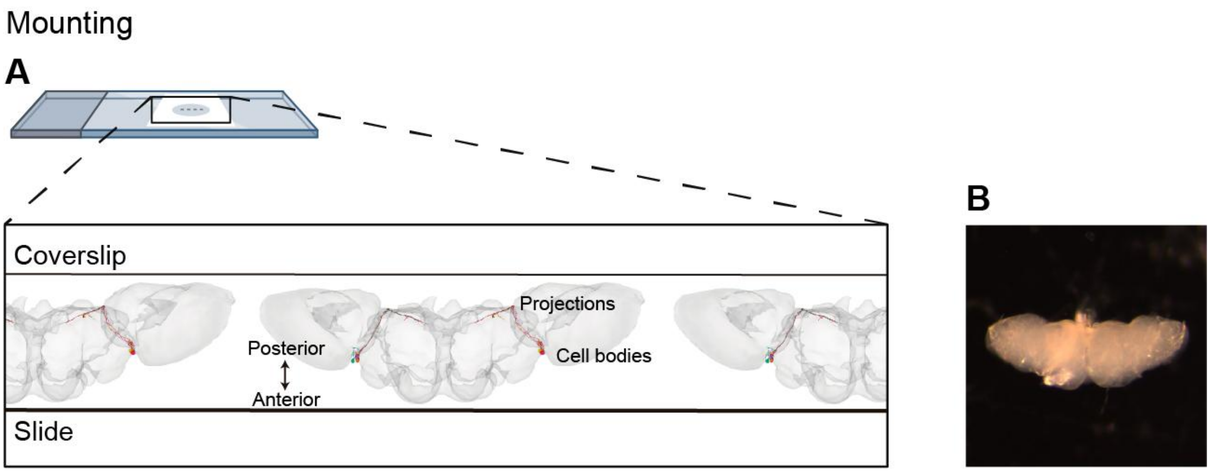

Supplementary Figure 4. Schematic diagram of the brain mounting orientation. (A) A cross-sectional view illustrates brain placement between the glass slide and coverslip. Tissue is oriented along the anterior-posterior axis, with the anterior surface resting on the slide and the posterior surface facing the coverslip (and the microscope objective). The relative locations of somatic cell bodies and their neuronal projections are indicated to highlight the optical advantages of this mounting strategy. Alternatively, brains can be mounted between two coverslips to allow for inversion of the preparation depending on imaging requirements. Images of the 3D reconstructed brains were captured from the FlyWire Connectome Data Explorer (2,3). **(B)** Image of a real brain oriented as shown in **(A)**.

	**Analysis**	**ANOVA**				**Contrast**				
		**Factor**	**df**	**F value**	**p value**	**Comparison**		**df**	**t ratio**	**p value**
**Figure 2**	**GFP signal (complexity)**	ZT	1; 273	0,100	0,753	**ZT2 vs ZT14**				
		Genotype		5,146	0,025	Left hemisphere	CD8::GFP	175	1,491	0,138
		Hemisphere		0,334	0,564		CD8::GFP + *Pdf*-RFP	173	-1,266	0,207
		ZT * Genotype		5,174	0,024					
		ZT * Hemisphere		0,050	0,823	Right hemisphere	CD8::GFP	179	1,213	0,227

		Genotype * Hemisphere		1,289	0,258		CD8::GFP + *Pdf*-RFP	156	-0,615	0,540
		Triple interaction		0,193	0,661					
	**RFP signal (complexity)**	ZT	1; 171	11,044	0,001	**ZT2 vs ZT14**				
		Genotype		23,733	< 0,001	Left hemisphere	*Pdf*-RFP	177	3,887	< 0,001
		Hemisphere		0,002	0,964		CD8::GFP + *Pdf*-RFP	171	-1,085	0,280
						Right hemisphere	*Pdf*-RFP	175	2,326	0,021
		ZT * Genotype		16,321	< 0,001		CD8::GFP + *Pdf*-RFP	156	-0,767	0,444
		ZT * Hemisphere		0,018	0,894	**CD8::GFP + *Pdf*-RFP vs *Pdf*-RFP**				
		Genotype * Hemisphere		2,298	0,131	Left hemisphere	ZT2	85.6	4,397	< 0,001
							ZT14	89	-0,451	0,0916
		Triple interaction		0,444	0,506	Right hemisphere	ZT2	85.6	4,512	< 0,001
							ZT14	89	1,705	0,6533
	**PDF signal (complexity)**	ZT	1; 257	31,536	< 0,001	**ZT2 vs ZT14**				
		Genotype	2; 257	27,785	< 0,001	Left hemisphere	CD8::GFP	248	2,005	0,046
		Hemisphere	1; 257	0,882	0,349		*Pdf*-RFP	268	4,918	< 0,001
		ZT * Genotype	2; 257	11,69	< 0,001		CD8::GFP + *Pdf*-RFP	244	-1,106	0,270
		ZT * Hemisphere	1; 257	0,094	0,7591	Right hemisphere	CD8::GFP	258	2,024	0,044

		Genotype * Hemisphere	2; 257	0,799	0,4509		*Pdf*-RFP	268	3,633	< 0,001
		Triple interaction	2; 257	0,352	0,7038		CD8::GFP + *Pdf*-RFP	219	-0,057	0,954
**Figure 3**	**Model selection**	ZT	1; 273	65,360	< 0,001	**ZT2 vs ZT14**				
		Temperature	2; 273	4,827	0,009	22°C	Low intensity	273	3,930	< 0,001
		Light intensity	1; 273	0,516	0,473		High intensity	273	3,605	< 0,001
		ZT * Temperature	2; 273	0,441	0,644	25°C	Low intensity	273	3,115	0,002
		ZT * Light intensity	1; 273	0,239	0,626		High intensity	273	2,588	0,010
		Temperature * Light intensity	2; 273	0,936	0,394	28°C	Low intensity	273	2,437	0,016
		Triple interaction	2; 273	0,796	0,452		High intensity	273	4,228	< 0,001
	**Additive model (ZT + Temperature)**	ZT	1	62,814	< 0,001	ZT2 vs ZT14		281	7,951	< 0,001
		Temperature	2	5,138	0,006	22°C vs 25°C		281	3.031	0,008
						22°C vs 28°C		281	2.409	0,044
						25°C vs 28°C		281	-0,572	0,835

Supplementary Table 1. Detailed statistical outputs corresponding to the data presented in Figures 2 and 3. Summary of all general linear models, including main effects, interactions, and post-hoc contrasts for terminal complexity across different genotypes, times of day (ZT), and environmental conditions (temperature and light intensity). Test statistics (F values for ANOVAs and t ratios for contrasts), degrees of freedom (df), and exact p-values are provided for each comparison.

	Parameter	Value
**Correlation analysis**	Pearson correlation coefficient (r)	0.8355
	95% CI	0.7567–0.8904
	R^2	0.6981
	p value	< 0.0001
	Sample size (n)	84
**Bland-Altman analysis**	Bias	2.950
	SD of bias	4.227
	95% limits of agreement (lower)	−5.336
	95% limits of agreement (upper)	11.24
	Sample size (n)	84

Supplementary Table 2. Method comparison using Pearson correlation and Bland-Altman analysis. Statistical parameters assessing the linear correlation (Pearson's r, R^2, and 95% CI) and absolute agreement (bias and 95% limits of agreement) between RFP and PDF signals for complexity quantification.

Variable	Fluoromount-G (n = 22)	Vectashield (n = 20)	Statistical Test	t	df	p-value
**Terminal length**	36.48 ± 1.359	49.18 ± 1.892	Student's t-test	5.53	40	< 0.0001
**Complexity**	24.47 ± 1.679	44.93 ± 2.734	Welch's t-test	6.38	31.93	< 0.0001

Supplementary Table 3. Terminal metrics in Fluoromount-G vs. Vectashield mounting media. Values represent Mean ± SEM. Statistical differences were assessed via Welch's t-test (complexity, XY spread) or unpaired Student's t-test (terminal length).

References

1. Petsakou A, Sapsis TP, Blau J. Circadian rhythms in rho1 activity regulate neuronal plasticity and network hierarchy. Cell. 2015 Aug 13;162(4):823–35.
2. Matsliah A, Sterling AR, Dorkenwald S, Kuehner K, Morey R, Seung HS, et al. Codex: Connectome Data Explorer. Unpublished. 2023;
3. Dorkenwald S, Matsliah A, Sterling AR, Schlegel P, Yu S-C, McKellar CE, et al. Neuronal wiring diagram of an adult brain. Nature. 2024 Oct 2;634(8032):124–38.
